# Vitamin A Deficiency Alters the Phototransduction Machinery and Distinct Non-Vision-Specific Pathways in the *Drosophila* Eye Proteome

**DOI:** 10.3390/biom12081083

**Published:** 2022-08-06

**Authors:** Mukesh Kumar, Canan Has, Khanh Lam-Kamath, Sophie Ayciriex, Deepshe Dewett, Mhamed Bashir, Clara Poupault, Kai Schuhmann, Oskar Knittelfelder, Bharath Kumar Raghuraman, Robert Ahrends, Jens Rister, Andrej Shevchenko

**Affiliations:** 1 Max Planck Institute of Molecular Cell Biology and Genetics, Pfotenhauerstraße 108, 01307 Dresden, Germany; 2 Department of Biology, University of Massachusetts Boston, Integrated Sciences Complex, 100 Morrissey Boulevard, Boston, MA 02125, USA; 3 Department of Analytical Chemistry, University of Vienna, 1090 Vienna, Austria

**Keywords:** *Drosophila*, retina, vitamin A, retinal, proteome, lipidome, phototransduction, mitochondrion

## Abstract

The requirement of vitamin A for the synthesis of the visual chromophore and the light-sensing pigments has been studied in vertebrate and invertebrate model organisms. To identify the molecular mechanisms that orchestrate the ocular response to vitamin A deprivation, we took advantage of the fact that *Drosophila melanogaster* predominantly requires vitamin A for vision, but not for development or survival. We analyzed the impacts of vitamin A deficiency on the morphology, the lipidome, and the proteome of the *Drosophila* eye. We found that chronic vitamin A deprivation damaged the light-sensing compartments and caused a dramatic loss of visual pigments, but also decreased the molar abundance of most phototransduction proteins that amplify and transduce the visual signal. Unexpectedly, vitamin A deficiency also decreased the abundances of specific subunits of mitochondrial TCA cycle and respiratory chain components but increased the levels of cuticle- and lens-related proteins. In contrast, we found no apparent effects of vitamin A deficiency on the ocular lipidome. In summary, chronic vitamin A deficiency decreases the levels of most components of the visual signaling pathway, but also affects molecular pathways that are not vision-specific and whose mechanistic connection to vitamin A remains to be elucidated.

## 1. Introduction

Vitamin A is an essential nutrient that is required in vertebrates and invertebrates for the synthesis of the retinylidene chromophore and the visual pigments [1,2,3,4]. Like mammals, *Drosophila* cannot synthesize vitamin A de novo; both the lack of dietary vitamin A sources [5,6,7] and the mutation of genes that are required for vitamin A synthesis [8,9] cause severe visual defects. Vitamin A is the precursor of the chromophore 11-*cis*-3-hydroxyretinal [10,11] that is required for the synthesis and function of the major visual pigment Rhodopsin 1 (Rh1) [6,12,13,14].

Beyond the well-studied visual pigment defect, the molecular impacts of vitamin A deprivation on the eye remain poorly understood. Chronic vitamin A deficiency is difficult to study in mammals because of vitamin A’s additional role in retinoic acid signaling that regulates diverse processes such as embryonic development, growth, and immunity [15]. In contrast, *Drosophila melanogaster* does not use canonical retinoic acid signaling [16,17,18] and predominantly requires vitamin A for vision [19]. The *Drosophila* eye is therefore an excellent model to study the molecular impacts of chronic vitamin A deficiency. A pioneering study [5] identified Rh1 as the first vitamin A-dependent *Drosophila* protein and detected additional proteins whose levels were decreased in the vitamin A-deficient eye. However, the identity of these proteins remained unknown.

**Figure 1:**
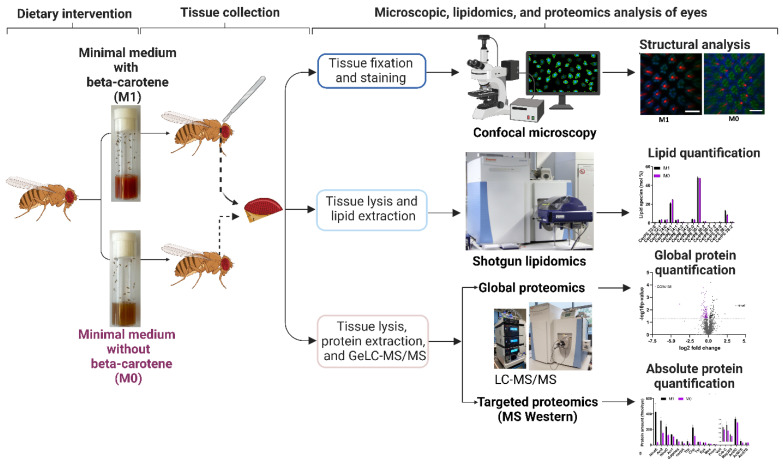
Experimental workflow to analyze the impacts of vitamin A deficiency on the morphology, the lipidome, and the proteome of the *Drosophila* eye. Schematics were created with BioRender. For details, see text.

To identify the molecules and pathways that respond to vitamin A deficiency in the *Drosophila* eye, we performed shotgun lipidomics [20,21] and GeLC-MS/MS for global proteomics [22] combined with MS Western [23] to quantify the absolute abundances of selected proteins that are critical for phototransduction and photoreceptor morphology (Figure 1). We found that vitamin A is required for the proper expression levels of the visual pigments and most phototransduction proteins, but also of components of major metabolic pathways such as the TCA cycle and the respiratory chain. In summary, our study revealed that vitamin A is critical for the expression of the visual signaling cascade, but also has non-vision-specific functions in the *Drosophila* eye that remain to be elucidated.

## 2. Materials and Methods

### 2.1. Food Media

*Drosophila* flies were raised on ‘standard’ lab diet (SF) or one of two synthetic diets (M0, M1). SF diet contained per liter: 8 g agar, 18 g brewer’s yeast, 10 g soybean, 22 g molasses, 80 g cornmeal, 80 g malt, 6.3 mL propionic acid, and 1.5 g nipagin. For both M0 and M1 diet, we dissolved 10 g of yeast extract (Kerry, Boston, MA, USA), 10 g of glucose (Merck, Branchburg, NJ, USA), and 1 g of UltraPure Agarose (Invitrogen, Waltham, MA, USA) in 100 mL of filtered tap water. The mixture was microwaved until it boiled and then cooled to 65–70 °C. Next, we added a solution of 0.1 g of stigmasterol (Sigma, St. Louis, MO, USA) in 2 mL of 95% ethanol and 1.5 mL of 10% nipagin (Sigma, St. Louis, MO, USA). In the case of M1 medium, we added a solution of 0.1 g beta-carotene (Sigma, St. Louis, MO, USA) in 2 mL of 95% ethanol. After thorough mixing for several minutes, the medium was poured into plastic vials (Genesee Scientific, El Cajon, CA, USA) and then allowed to solidify at room temperature.

### 2.2. Drosophila Melanogaster Stocks

The *Drosophila melanogaster* wild-type strains Oregon R and Canton S were raised on the respective food medium from the embryonic to the adult stage under a 12 h light/12 h dark cycle at 25 °C. Three to four days old male flies were used for all experiments.

### 2.3. Imaging the Drosophila Eye

Adult flies were immobilized by embedding them in an agarose gel. To prepare the agarose gel, we dissolved 2 g of UltraPure Agarose (Invitrogen, Waltham, MA, USA) in 100 mL distilled water. The solution was heated in a 500 mL Erlenmeyer flask for several minutes until bubbles were visible. The flask was then placed in a water bath (Thermo Scientific, Waltham, MA, USA) set to 58 °C to cool the solution down to 58 °C. Next, flies were anesthetized with carbon dioxide gas and transferred to a 60 mm petri dish (Corning, Corning, NY, USA) filled with about 10 mL of the liquid agarose gel. We then submerged the wings and legs using forceps and placed the petri dish on ice for the gel to solidify. After solidification of the gel, the petri dish was placed under a Stemi 508 Trinoc microscope (model #4350649030, Zeiss, White Plains, NY, USA), and the fly head was adjusted with the forceps such that one eye was oriented in parallel to the microscope lens. The petri dish was placed back on ice until imaging, which was performed with an Axiocam 208 HD/4k color camera (model #4265709000) set to auto exposure and auto white balance. Images were processed with Fiji (https://imagej.net/software/fiji/ accessed on 10 July 2022) [24], Adobe Photoshop 2020, and Adobe Illustrator 2020 software.

### 2.4. Confocal Microscopy and Immunohistochemistry of Drosophila Photoreceptors

We dissected retinas of four days old flies in cold PBS, as previously described [25]. We fixed the retinas in 3.8% formaldehyde solution before removing the laminas and the head cuticle. We then incubated the retinas overnight in the primary antibodies (mouse anti-Rh1 4C5, 1:10, from Developmental Studies Hybridoma Bank, University of Iowa; rabbit anti-Rh6, 1:2000, a gift from Claude Desplan, New York University) that were diluted in PBST (PBS + 0.3% Triton-X, Sigma, St. Louis, MO, USA). We removed the primary antibody solution the next morning and washed the retinas three times with PBST. In the evening, we incubated the retinas in the secondary antibodies diluted in PBST (1:800, Alexa Fluor 555- and 647-conjugated raised in donkey; Invitrogen, Waltham, MA, USA) and Alexa Fluor 488-conjugated Phalloidin (1:100; Invitrogen, Waltham, MA, USA). We performed three PBST washes the next morning, mounted the retinas using SlowFade (Molecular Probes, Eugene, OR, USA) on a bridge slide, and imaged them with a Zeiss LSM 8 confocal microscope. We processed the raw images with Fiji and further cropped and contrasted them with Adobe Photoshop 2020 and Adobe Illustrator 2020.

### 2.5. Rhabdomere Measurements and Statistics

We used Fiji’s measurement tool on the Phalloidin channel to quantify the cross-sectional area of the rhabdomeres of five ommatidia from five different adult retinas. We used the Rh6 antibody channel to reproducibly perform quantifications at the level of the Rh6-positive stalk of the R8 photoreceptors. We used the freehand selection tool to draw a circle along the circumference of an R3 or an R8 photoreceptor rhabdomere, respectively. Next, we used the ROI manager tool to measure the area of the circled rhabdomere cross-section. Finally, we used RStudio (https://www.rstudio.com/ accessed on 10 July 2022) to generate Box-and-Whisker plots and to perform an ANOVA followed by a Tukey’s HSD test to determine whether the measured areas were statistically significant between different dietary groups. The significance cut-off was *p* < 0.05.

### 2.6. Lipid Extraction and Shotgun Lipidomics on the Drosophila Eye

Lipid analysis was performed as previously described [26,27]. Briefly, whole adult eyes (N = 10) were separated with a thin blade and placed in 40 µL of 150 mM ammonium bicarbonate buffer containing 10% of isopropanol in a 2 mL Eppendorf tube, snap-frozen in liquid nitrogen, and stored at −80 °C or processed immediately. Since we used whole eyes, the samples contained photoreceptor neurons as well as other cell types such as pigment cells, lens-secreting cone cells, and bristle cells. The eyes were mechanically disrupted with 1 mm zirconia beads and the samples were dried under vacuum to remove isopropanol. Lipids were recovered using methyl-*tert*-butyl ether (MTBE) extraction [28]. Samples were resuspended in 200 µL of water and 700 µL of MTBE/methanol (5:1.5, *v*/*v*) containing internal standards (0.539 nmol zymosterol-d5, 0.782 nmol stigmasterol-d6, 0.313 nmol triacylglycerol-d5 50:0, 0.073 nmol diacylglycerol-d5 34:0, 0.138 nmol PC 25:0, 0.109 nmol LPC 13:0, 0.067 nmol PS 25:0, 0.147 nmol PE 25:0, 0.053 nmol LPE 13:0, 0.090 nmol PI 25:0, 0.068 nmol PG 25:0, 0.102 nmol Cer 30:1, 0.077 nmol PA 25:0, 0.068 nmol GalCer 30:1, 0.081 nmol LacCer 30:1, 0.074 nmol CerPE 29:1). After centrifugation, the upper phase was collected and dried under vacuum. The entire extraction procedure was performed at 4 °C. Prior to sterol analysis, derivatization with acetyl chloride was performed as previously described [29]. Otherwise, the total lipid extracts were diluted and infused into a mass spectrometer using the robotic nanoflow ion source TriVersa NanoMate (Advion BioSciences, Ithaca, NY, USA). Shotgun analyses were performed on a Q Exactive hybrid tandem mass spectrometer (Thermo Fisher Scientific, Bremen, Germany). FTMS and DIA FTMS/MS spectra in positive and negative mode were acquired at the mass resolution R _*m*/*z* = 200_ = 140.000 (full width at half maximum, FWHM). Lipids were identified and quantified using the LipidXplorer software [30,31].

### 2.7. MS Western and Global Proteome Analysis of the Drosophila Eye by GeLC-MS/MS

Adult eyes (N = 40) were separated from the heads and placed in a lysis buffer containing 150 nM NaCl, 1 mM EDTA, 50 mM Tris-HCl (pH 7.5), 1 tablet Roche protease inhibitors, 0.2% *w*/*v* CHAPS, 0.1% *w/v* OGP, 0.7% *v*/*v* Triton X-100, as well as 0.25 μg/mL DNAse and RNAse. The samples were immediately snap frozen with liquid nitrogen and stored at −80 °C or processed immediately. The eye tissues were homogenized and an equal volume of 2X SDS Laemmli sample buffer (SERVA Electrophoresis GmbH, Heidelberg, Germany) was added to the supernatant. The samples were incubated at 80 °C for 10–15 min and loaded on 4–20% 1D SDS PAGE. The protein bands were visualized by Coomassie Brilliant Blue R250 staining. Each gel lane was cut into six gel slices and each gel slice was co-digested with heavy isotope-labeled MS Western protein standard and a gel band containing 1 pmol of BSA standard. Details of the MS Western method and the MS western protein standard used in this study have been previously described [23,32].

In-gel digestion was carried out as previously described [33]. Briefly, the electrophoresed gel was rinsed with water and stained with Coomassie Brilliant Blue R250 for 10 min at room temperature, and then destained with a mixture of water:methanol:acetic acid (50:40:10 *v*/*v*/*v*). Each gel slice was cut into small pieces of about 1 mm size. The gel pieces were then transferred into 1.5 mL LoBind Eppendorf tubes and completely destained by acetonitrile (ACN)/water. Reduction was done by incubating the gel pieces with 10 mM dithiothreitol at 56 °C for 45 min. Alkylation was carried out with 55 mM iodoacetamide for 30 min in the dark at room temperature. The reduced and alkylated gel pieces were washed with water/ACN and finally shrunk with ACN; ice cold trypsin (10 ng/µL) was added to cover the shrunken gel pieces and after 1 h of incubation on ice, excess trypsin (if any) was discarded. The gel pieces were then covered with 10 mM NH_4_HCO_3_ and incubated for 12–15 h at 37 °C. The tryptic peptides were extracted using water/ACN/formic acid, dried using a vacuum centrifuge, and stored at −20 °C until next use. The tryptic peptides were recovered in 5% aqueous formic acid, and 5 μL were injected using an autosampler into a Dionex Ultimate 3000 nano-HPLC system, equipped with a 300 μm i.d. × 5 mm trap column and a 75 μm × 15 cm Acclaim PepMap100 C18 separation column. The 0.1% formic acid in water and ACN were used as solvent A and B, respectively. The samples were loaded on the trap column for 5 min with a solvent A flow of 20 µL/min. The trap column was then switched online to the separation column, and the flow rate was set to 200 µL/min. The peptides were fractionated using a 180 min elution program: a linear gradient of 0% to 30% B was delivered in 145 min and then B% was increased to 100% within 10 min, maintained for another 5 min, dropped to 0% in 10 min, and maintained for another 10 min. Mass spectra were acquired in data-dependent mode on a Q Exactive HF mass spectrometer (Thermo Fisher Scientific, Bremen, Germany); the experimental settings are provided in Table S3.

### 2.8. Data Processing for Protein Identification and Quantification

Mascot v2.2.04 (Matrix Science, London, UK) was used for peptide identifications against the *Drosophila* reference proteome database from UniProt (https://www.uniprot.org/ accessed on 10 July 2022) to which sequences of human keratins and porcine trypsin were added. The database searches were performed with the following settings: precursor mass tolerance of 5 ppm; fragment mass tolerance of 0.03 Da; fixed modification: carbamidomethyl (C); variable modifications: acetyl (protein N-terminus), oxidation (M); in MS Western experiments, Label: 13C (6) (K), Label: 13C (6) 15N (4) (R), up to 2 missed cleavages were allowed. Progenesis LC-MS v4.1 (Nonlinear dynamics, UK) was used for the peptide feature extraction and the raw abundance of identified peptides was used for absolute quantification. MaxQuant v1.5.5.1 (https://maxquant.org/ accessed on 10 July 2022) and Perseus v1.5.5.3 (https://maxquant.net/perseus/ accessed on 10 July 2022) were used for label-free quantification and subsequent statistical analysis. MaxQuant analysis was done under default settings. Protein annotations, including their transmembrane properties, were fetched from AnnotationDB package (version 1.48.0) using R (version 3.6).

### 2.9. Functional Annotation and Enrichment Analysis

We performed a pathway enrichment analysis to identify the significantly dysregulated pathways and proteins. The analysis was done using the ClueGO plugin (version 2.5.7) [34] on the open-source network visualization platform Cytoscape (version 3.8.0) (https://cytoscape.org/ accessed on 10 July 2022). The Kyoto Encyclopedia of Genes and Genomes (KEGG) (release 93) (https://www.genome.jp/kegg/ accessed on 10 July 2022) and Reactome databases (release 71) (https://reactome.org/ accessed on 10 July 2022) of *Drosophila melanogaster* were downloaded. The *p*-value of each pathway was computed using a hypergeometric test and corrected by applying the Benjamini-Hochberg procedure. Pathway enrichment was performed on gene identifiers of the queried proteins. In this step, we applied two criteria: (1) an enriched pathway must include at least three genes of protein query list and the matching genes can cover minimum 4% of the annotated genes of a pathway, and (2) at least one of the found proteins must be significantly regulated. Missing parent nodes were added externally based on the hierarchy of the corresponding pathway database. The coverage of each pathway was computed and the sum of log2FC of mapped up- and downregulated proteins separately represented the regulation value of each pathway. Gene Ontology (GO) term enrichment analysis on cellular components was performed using the GOrilla algorithm [35] and the representative terms of redundant GO-Terms were found by using the ReviGO algorithm [36].

## 3. Results 

### 3.1. Wild Type Eye and Rhabdomere Morphology of Drosophila Raised on a Minimal Synthetic Diet

The goal of our study was to analyze the molecular response of the *Drosophila* eye to vitamin A deprivation. To this end, we established a minimal synthetic diet to manipulate the vitamin A content. This synthetic diet consisted of a water-soluble yeast extract, agar, glucose, and nipagin (see Materials and Methods). Since *Drosophila* cannot synthesize sterols [37], which it requires for the synthesis of biological membranes and ecdysteroid hormones, we added the plant sterol stigmasterol that effectively supports *Drosophila* development from the larval to the adult stage [26]. Because yeast does not provide significant sources of vitamin A [7], the use of a water-soluble yeast extract as the basis for the synthetic medium allowed us to manipulate the levels of vitamin A by either adding (M1 diet) or omitting (M0 diet) its precursor beta-carotene, respectively (Figure 1). To determine whether the vitamin A-providing M1 control diet supported wild type eye and photoreceptor development, we compared it to a nutrient-rich ‘standard’ lab food (SF) that contained commonly used natural ingredients [38] (see Materials and Methods).

**Figure 2:**
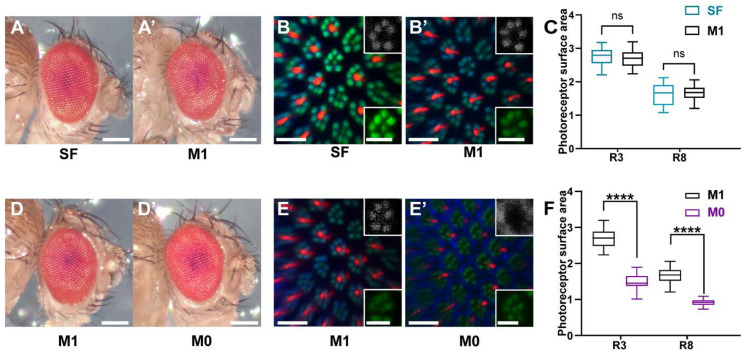
Eye and rhabdomere morphology of wild type *Drosophila melanogaster* raised on nutrient-rich ‘standard’ lab diet (SF), synthetic M1 diet containing a vitamin A precursor, or synthetic M0 diet without vitamin A precursors. (**A**,**A’**) Compound eyes of wild type flies that were raised on a ‘standard’ lab diet (SF) or a minimal synthetic diet (M1). Scale bars, 250 µm. (**B**,**B’**) Adult retina confocal cross-sections of wild type flies raised on SF or M1 diet. Seven F actin-rich (Phalloidin, green) rhabdomeres are visible in each unit eye (green channel); Rh1 (blue and gray channel) is expressed in the rhabdomeres of ‘outer’ photoreceptors and Rh6 (red) is expressed in the rhabdomeres of ‘inner’ photoreceptors. Scale bars, 10 µm (insets, 5 µm). (**C**) Quantification of the cross-sectional areas of the rhabdomeres (n = 25 for each condition) for two photoreceptor types (R3: ‘outer’ photoreceptor; R8: ‘inner’ photoreceptor) of flies raised on SF or M1 diet. ns = not significant. (**D**,**D’**) Compound eyes of wild type flies that were raised on M1 control diet or M0 diet that lacks vitamin A precursors. Scale bars, 250 µm. (**E**,**E’**) Adult retina confocal cross-sections of wild type flies raised on M1 or M0 diet. Seven circular rhabdomeres (Phalloidin, green) are present in each unit eye (green channel). Rh1 (blue and gray channel) is specifically expressed in the rhabdomeres of ‘outer’ photoreceptors and Rh6 (red) is expressed in the rhabdomeres of ‘inner’ photoreceptors in the case of M1 diet. Note the reduced rhabdomere cross-sectional area (green) as well as the abnormal accumulation of Rh1 (blue and gray) and Rh6 (red) outside of the rhabdomeres in the case of M0 diet. Scale bars, 10 µm (insets, 5 µm). (**F**) Quantification of the cross-sectional areas of the rhabdomeres (n = 25 for each condition) of two different photoreceptor types (R3: ‘outer’ photoreceptor; R8: ‘inner’ photoreceptor) for M1 and M0 diet.

Wild type flies that were raised on nutrient-rich SF diet or minimal synthetic M1 diet showed a normal external eye morphology (Figure 2A,A’). Moreover, immunohistochemistry and confocal microscopy revealed a wild type morphology of the light-sensing compartments (rhabdomeres) of the rod-equivalent ‘outer’ photoreceptors and the cone-equivalent ‘inner’ photoreceptors [39] for both diets (Figure 2B,B’). The spatial expression patterns of the major Rhodopsin Rh1 in the ‘outer’ rhabdomeres and of Rh6 in the ‘inner’ rhabdomeres were also wild type (Figure 2B,B’). Lastly, the quantification of the cross-sectional areas of the rhabdomeres of both photoreceptor types (R3: ‘outer’ photoreceptor; R8: ‘inner’ photoreceptor) revealed no significant difference between the two dietary conditions (Figure 2C). Taken together, these data suggest that the minimal synthetic M1 diet supports normal eye and rhabdomere morphology and can thus be used to analyze the impacts of dietary manipulations on the eye.

### 3.2. Vitamin A Deficiency Affects Rhabdomere Volume and Rhodopsin Expression

To study the morphological consequences of vitamin A deficiency, we compared vitamin A replete (M1 diet) eyes with vitamin A-deficient eyes (M0 diet). Chronic vitamin A deprivation of wild type flies did not affect the external morphology of the eye (Figure 2D,D’). However, the visual pigments Rh1 and Rh6 accumulated abnormally outside of the rhabdomeres in vitamin A-deficient eyes (Figure 2E,E’). Moreover, the cross-sectional area of the membrane-rich rhabdomeres was dramatically reduced (Figure 2E,E’) for both the ‘inner’ and the ‘outer’ photoreceptor types (median 46% reduction for R3 and 45% for R8; *p* < 0.001) compared to flies that were raised on vitamin A replete M1 diet (Figure 2F). Taken together, vitamin A deprivation did not affect the external eye morphology but caused an abnormal localization of the Rhodopsins outside of drastically reduced rhabdomeres.

### 3.3. Vitamin A Deficiency Has No Apparent Effects on the Lipidome of the Drosophila Eye

To analyze whether the severely damaged membrane-rich rhabdomeres in vitamin A-deficient eyes were the result of a perturbed ocular membrane lipid composition, we compared the lipidomes of vitamin A replete and vitamin A-deficient eyes. We performed shotgun lipidomics [20,21] on total lipid extracts from whole adult eyes (N = 10 for each dietary condition) and quantified the molar abundance of more than 250 lipid species from 13 major lipid classes (Figure 1 and Table S1). Shotgun lipidomics covered major classes of membrane lipids and energy storage lipids, while signaling lipids (e.g., PIP2) were not identified because of their very low abundance. Given that we analyzed whole retinas, it is possible that potential photoreceptor-specific lipid differences were masked by the (few) other cell types of the retina.

**Figure 3:**
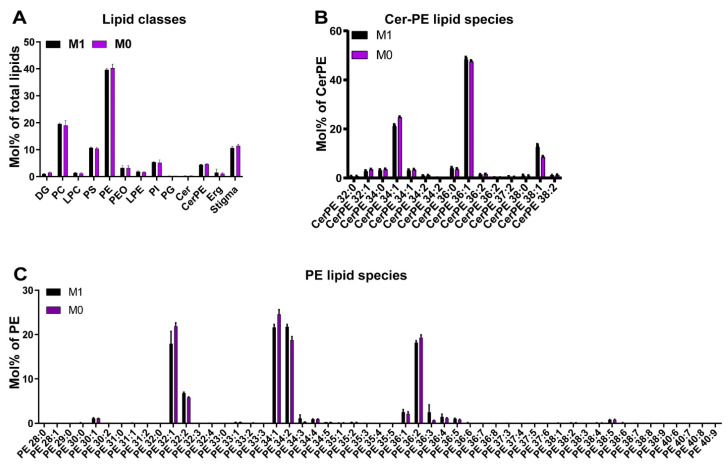
The eye lipidome of wild type *Drosophila melanogaster* raised on synthetic M1 diet (contains vitamin A precursor) or synthetic M0 diet (lacks vitamin A precursors). (**A**) Quantification (in mol%) of different lipid classes in the wild type *Drosophila* eye for M1 diet (control, black) and M0 diet (vitamin A deficiency, purple). (**B**) Analysis of ocular phospatidylethanolamine ceramide (Cer-PE) lipid species for M1 diet (black) and M0 diet (purple). (**C**) Analysis of ocular phospatidylethanolamine (PE) lipid species for M1 diet (black) and M0 diet (purple).

The eyes of chronically vitamin A-deprived flies did not show any significant changes in lipid composition (Figure 3A–C). Both the lipid class composition and molecular species profiles of major membrane constituents, such as sphingolipid and glycerophospholipid classes, were consistent with previous reports [7,26]; for instance, the phosphatidylethanolamines (PE), phosphatidylcholines (PC), and phosphatidylinositols (PI) were enriched with saturated and mono-unsaturated species that are typical for flies that are reared on yeast-based diets (Figure 3B,C). The relative abundances of the lipid classes were also comparable between both dietary conditions: PE was the most abundant lipid class with about 40 mol% of the total lipids, followed by PC that was close to 20 mol%, and then phosphatidylserines (PS) as well as sterols that were each about 10 mol% (Figure 3A). As expected, the supplemented phytosterol stigmasterol was the predominant sterol in both groups (Figure 3A). Taken together, vitamin A deficiency had no apparent effects on the ocular lipidome.

### 3.4. Vitamin A Deficiency Alters the Phototransduction Machinery 

**Figure 4:**
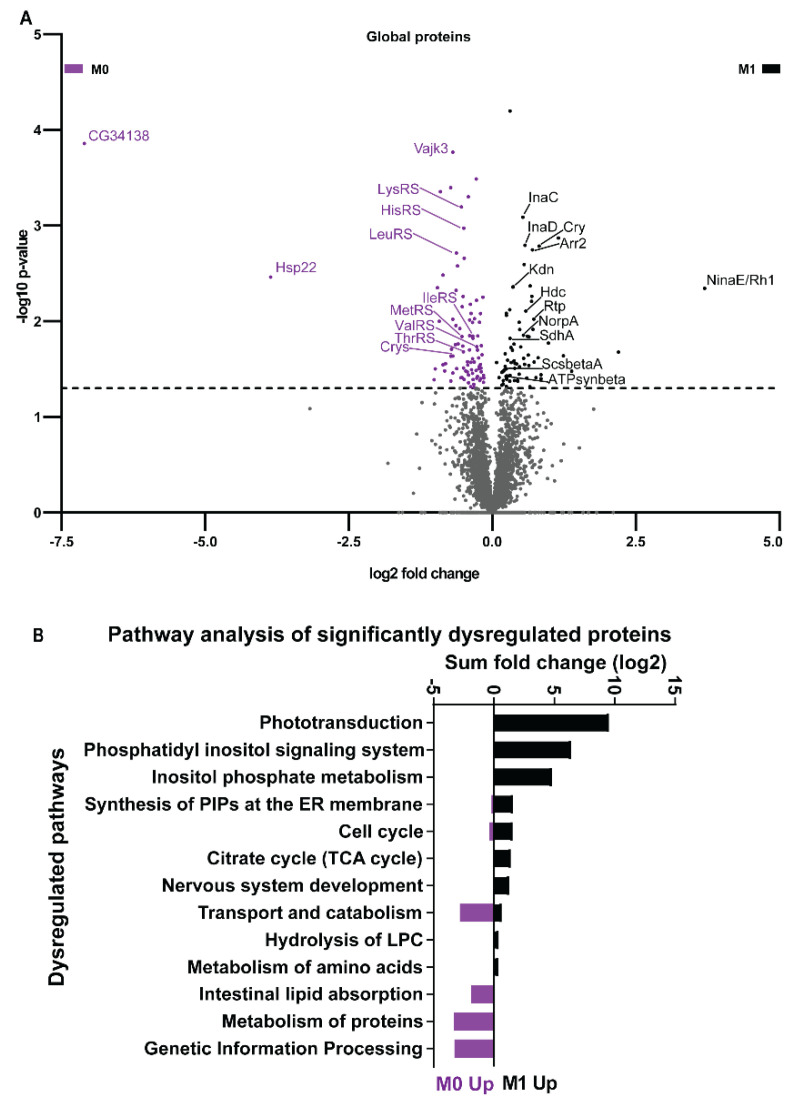
The eye proteome of wild type *Drosophila melanogaster* raised on synthetic M1 diet (contains vitamin A precursor) or synthetic M0 diet (lacks vitamin A precursors). (**A**) Label-free global eye proteome analysis. The volcano plot shows differentially expressed proteins between M1 diet (black) and M0 diet (purple) in the *Drosophila* eye. The statistical cut-off (*p*-value -log10 1.3, equivalent to *p* < 0.05) is indicated by the dashed line. (**B**) Pathway enrichment analysis of differentially expressed proteins in the eyes of flies raised on M1 diet or M0 diet. Note that the pathways ‘phototransduction’ and ‘citrate cycle’ were significantly downregulated in the absence of vitamin A, while ‘metabolism of proteins’ was upregulated.

Next, we analyzed the impact of vitamin A deficiency on the ocular proteome. We extracted total protein from adult eyes (N = 40 for each dietary condition) and performed label-free quantitative proteomics by GeLC-MS/MS [22,23] to quantify a total of 3281 proteins (Table S1). The relative quantification of protein abundances for both dietary conditions revealed a significant increase of 88 proteins and a significant decrease of 73 proteins in vitamin A-deficient eyes (Figure 4A and Table S1). A bioinformatic analysis of the significantly dysregulated proteins (Figure 4B and Table S1) showed that vitamin A deficiency significantly (*p* ≤ 0.05) downregulated components of the pathways ‘phototransduction’, ‘phosphatidylinositol/PI signaling’, and ‘citrate/TCA cycle’. Conversely, vitamin A deficiency upregulated components of ‘genetic information processing’, ‘transport and catabolism’, and ‘protein metabolism’ (Table 1).

**Figure 5:**
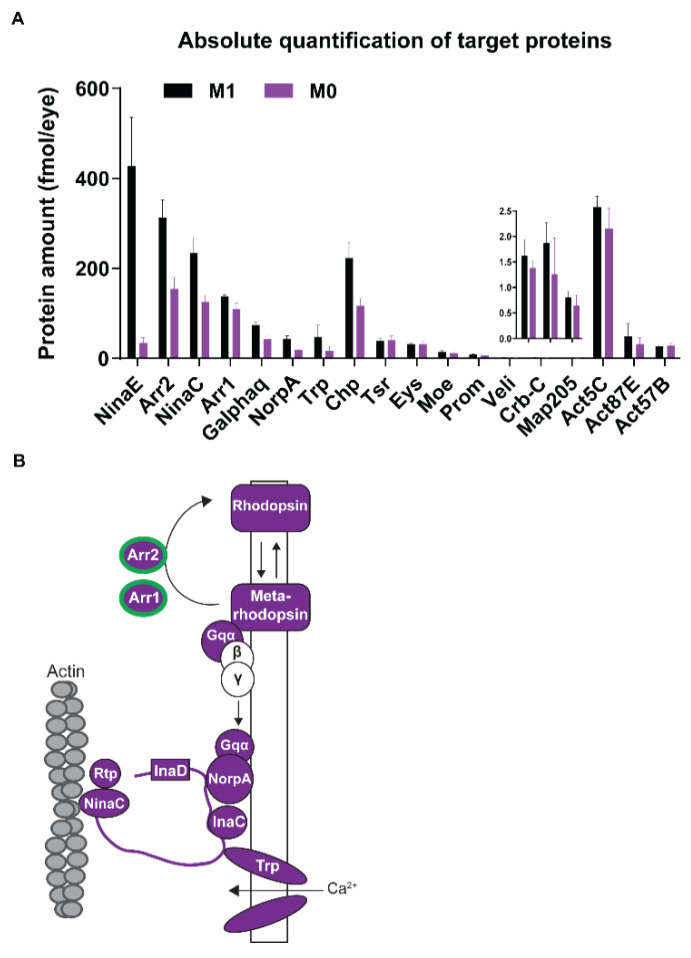
Quantification of major photoreceptor proteins of wild type *Drosophila melanogaster* raised on synthetic M1 diet or synthetic M0 diet (lacks vitamin A precursors). (**A**) Targeted absolute quantification of the molar amounts of proteins that play a major role in phototransduction or photoreceptor morphology for M1 diet (black) and M0 diet (purple), N = 3 for each condition. Note the reduced levels of most phototransduction proteins and the structural protein Chp in vitamin A-deficient eyes (purple). Error bar indicates mean ± SD. (**B**) Vitamin A deficiency significantly decreases the abundances of phototransduction proteins. Note that almost all components are significantly decreased (purple color); *Arr1* and *Arr2* are regulated on the transcriptional level (green outline).

Since the pathway ‘phototransduction’ was significantly downregulated in vitamin A-deficient eyes, we performed a global analysis of the relative abundance of specific phototransduction proteins. Consistent with previous Western blot results [13,14], we detected a substantial decrease of Rh1/NinaE in vitamin A-deficient eyes (Figure 4A and Figure 5A, and Table S2). Unexpectedly, we found that the levels of most downstream phototransduction proteins [40] were also significantly lower (Figure 4A): the scaffolding protein InaD [41] as well as its binding partners NorpA [42], the eye-specific kinase InaC [43], and the unconventional myosin NinaC [44] were all significantly decreased (Figure 4A and Figure 5A, and Table S2). In addition, NinaC’s interaction partners, the phosphoprotein Retinophilin (Rtp) [45,46] and the major Arrestin Arr2 [47], were also significantly reduced. Moreover, the circadian regulator Cry, which interacts with NinaC and InaD to modulate visual responses [48], was significantly decreased (Figure 4A and Table S2). Lastly, the enzyme Histidine decarboxylase (Hdc), which generates the neurotransmitter histamine that is released from the photoreceptors in response to light [49,50], was also significantly reduced in vitamin A-deficient eyes (Figure 4A and Table S2). Taken together, vitamin A deficiency decreased the levels of the visual pigment, most phototransduction proteins, and the enzyme that generates the photoreceptor neurotransmitter.

Our global proteome analysis yielded relative (fold-) changes of proteins in an unbiased manner but not their actual (molar) abundances, which would allow us to analyze the effects of vitamin A deprivation on the quantitative relationships of phototransduction proteins that interact in specific ratios. We therefore used MS Western [23] (Figure 1) to perform a targeted quantification of the stoichiometry of the major visual pigment Rh1 (NinaE) and the two visual Arrestins (Figure 5A,B, Table S2). Rh1 (mean content: 428 fmoles/eye) and the major Arrestin Arr2 (313 fmoles/eye), which interact with activated Rh1 and terminates visual signaling [47], were the most abundant visual signaling-related photoreceptor proteins in vitamin A replete eyes. The molar abundance of the minor Arrestin Arr1 [51], which mediates Rh1 endocytosis, was lower (138 fmoles/eye). Vitamin A deficiency drastically (by as high as 12.4-fold) reduced the abundance of Rh1 (to 34.47 fmoles/eye; Figure 5A and Table S2), which was the most dramatic reduction of all visual signaling-related proteins that we quantified. The decrease of the two Arrestins was much less severe, about 2-fold for Arr2 (to 155 fmoles/eye) and 1.3-fold for Arr1 (to 109.7 fmoles/eye). Hence, the molar ratios of neither Rh1:Arr1/Arr2 nor Arr1:Arr2 were preserved in vitamin A-deprived, Rh1-deficient eyes. However, the relative ranking of the phototransduction proteins according to their molar abundances was preserved (e.g., Arr2 was most abundant and NorpA was least abundant) because most of them showed a similar decrease in their molar content in vitamin A-deficient eyes (Figure 5B and Table S2).

Since vitamin A deficiency severely damaged the rhabdomeres (see above), we also quantified the molar content of major structural proteins that are required for photoreceptor morphology and maintenance [52]. In vitamin A replete eyes, their molar concentrations (Figure 5A) were consistent with the extent of the spatial expression patterns (broad or restricted): the cell adhesion molecule Chaoptin (Chp), which is broadly expressed throughout the perimeter of the rhabdomeres [53,54], was most abundant (Figure 5A). The molar concentration of structural proteins with restricted expression patterns—Eyes shut (Eys/Spam, located in the interrhabdomeral space) [54], Prominin (Prom, rhabdomere stalk and tips of the microvilli) [54], and Crumbs (Crb, rhabdomere stalk) [55]—were 10- to 20-fold less abundant (Figure 5A). However, in vitamin A-deficient eyes, the molar abundances of Chp (1.9-fold, 117 fmoles/eye; Figure 5A) and Prom (1.5-fold, 6.3 fmoles/eye) were significantly decreased, while Eys, Crb, and also three actins (Act5C, Act87E, Act57B) were unchanged (Figure 5A). Since we analyzed whole retinas, it is possible that such ubiquitously expressed proteins did not appear to be significantly changed because their unaffected abundance in non-photoreceptor cell types might have masked moderate changes in photoreceptor neurons. Taken together, the absolute quantification of major photoreceptor proteins not only validated the global proteome data but also revealed that the molar ratios of the functionally related phototransduction proteins and specific structural proteins were disrupted in the vitamin A-deficient eye. While the chronic lack of vitamin A affected almost all components of the phototransduction cascade (Figure 5B), it affected only specific photoreceptor morphology proteins.

In addition to phototransduction, phosphatidylinositol (PI) signaling was another vision-related pathway [56] that was altered by vitamin A deficiency (Figure 4B): it reduced the levels of NorpA that hydrolyses the membrane phospholipid PIP2 to the second messengers DAG and IP3 [57] (Table S1). Moreover, NorpA’s inhibitor InaC [58,59] was also less abundant in vitamin A-deficient eyes. The kinase PI4KIIIalpha that converts PI to the PIP2 precursor PI4P and thereby supports PIP2 levels during NorpA signaling [60], as well the scaffolding protein Ttc7 that is required for PiK4IIIalpha function [61], were both downregulated. Lastly, the PI-3-phosphatase Myotubularin-related protein 6 (Mtmr6/CG3530) [62], was also decreased in abundance. In summary, vitamin A deficiency decreased the levels of proteins that mediate the key steps of visual signaling in photoreceptors: the detection of light, phototransduction, PI signaling, and synaptic transmission.

### 3.5. Vitamin A Deficiency Alters Non-Vision-Specific Pathways of the Drosophila Eye Proteome

**Figure 6:**
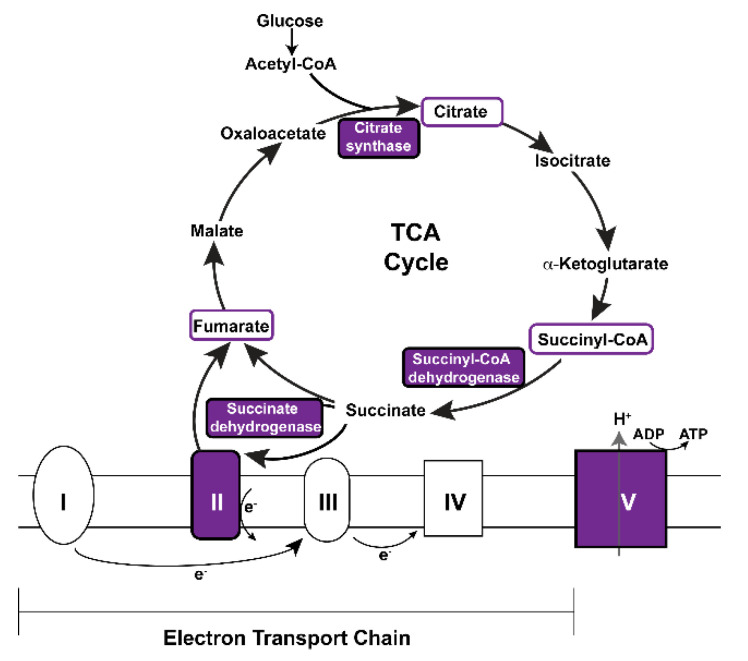
Vitamin A deficiency decreases the abundances of key components of the mitochondrial TCA cycle and electron transport chain. Purple color indicates significantly decreased proteins or subunits in vitamin A-deficient eyes. For details, see text.

Strikingly, our global proteome analysis enriched vitamin A-dependent pathways whose functions are not specific to visual signaling (Figure 4B, Table 1). For instance, this includes a decrease of components of major mitochondrial processes such as the citrate/TCA cycle and the electron transport chain [63]: vitamin A deficiency decreased the abundances of the succinyl-coenzyme A synthetase beta subunit ScsbetaA, the succinate dehydrogenase subunit A SdhA, the citrate synthase Kdn, and the ATP synthase beta subunit ATPsynbeta (Figure 4A and Figure 6, Table S1). In addition, nine other mitochondria-related proteins were also decreased (MRRF1, MRpS30, Alr, Marf, Cmpk, Retm, Men-b, CG7430, CG1635) (Table S1).

Conversely, we identified non-vision-specific proteins whose abundances increased in response to vitamin A deficiency. For instance, the predicted transmembrane protein CG34138 was the most increased (137-fold) protein (Figure 4A). Furthermore, several members of the category ‘transport and catabolism’ were upregulated: the cholesterol trafficking protein homolog Npc1a, the clathrin binding protein Gga, the lysosomal enzyme Ppt1, and the beta3-adaptin subunit Rb. In addition, we detected a significant increase in the levels of seven heat shock proteins and chaperones (Hsp22, Hsp23, Hsc70-3, Cnx99A, Gp93, CCT5, Wbl) as well as seven aminoacyl-tRNA synthetases (LeuRS, LysRS, MetRS, ThrRS, HisRS, IleRS, ValRS) (Figure 4A and Table S1). These protein folding- and translation-related proteins might be upregulated as a result of an unfolded protein response to impaired Rh1 synthesis (see Discussion) in the vitamin A-deficient photoreceptors.

Lastly, we detected a significant increase in the levels of ten cuticle-related and chitin binding-related proteins (Crys, Cpr62Bc, CG8192, Vajk3, CG42323, Gasp, CG7214, Acp1, Dpy, Slf) (Figure 4A and Table S1). Notably, the chitin-binding protein Vajk3 [64] is the second-most significantly upregulated protein in response to vitamin A deficiency. Moreover, the significantly upregulated Drosocrystallin (Crys) is a major lens protein [65,66]. Since the corneal lens of the *Drosophila* eye is a cuticular structure, this suggests that vitamin A deficiency affects protein expression in the lenses of the unit eyes. 

**Figure 7:**
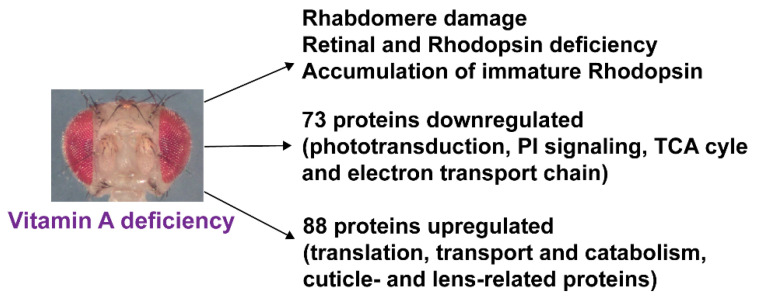
Summary of the morphological and molecular impacts of vitamin A deficiency on the *Drosophila melanogaster* eye. For details, see text.

In summary, vitamin A deficiency caused severe photoreceptor morphology defects and impaired the synthesis of the visual pigments. It did not have any apparent effects on the ocular lipidome but decreased the levels of major components of two visual signaling-related pathways (phototransduction and PI signaling) and the mitochondrial TCA cycle/electron transport chain. Conversely, vitamin A deficiency increased the abundances of proteins involved in translation and cuticle/chitin-related functions (Figure 7).

## 4. Discussion

Vitamin A deficiency damages the light-sensing compartments of the photoreceptors, impairs the synthesis of the visual chromophore and the visual pigments, and causes severe visual defects [67] (Figure 7). The goal of our study was to identify the molecular pathways in the *Drosophila* eye that respond to vitamin A deficiency. We found that a subset of the differentially expressed proteins has photoreceptor-specific functions, such as phototransduction, but most of the vitamin A-dependent proteins have roles that are not vision-specific. This strongly suggests that, like in mammals, vitamin A has important functions in *Drosophila melanogaster* beyond light detection, which is mediated by its metabolite retinal. It is possible that vitamin A (retinol) [68] or its other bioactive metabolite retinoic acid [69,70] have physiological functions in the *Drosophila melanogaster* eye.

### 4.1. Vitamin A Deficiency Decreases the Expression of Most Phototransduction Proteins

We discovered that vitamin A is not only required for the expression of the visual pigment Rh1, but also for most downstream phototransduction proteins (Figure 5B). Our previous [2] and current data suggest that their regulation involves vitamin A-dependent transcriptional as well as post-transcriptional mechanisms. A feedback mechanism decreases the expression of both *arrestin* genes in response to vitamin A deficiency [2] and we showed in the current study that this is consistent with the reduced protein levels of both visual Arrestins Arr1 and Arr2. The Arr1 and Arr2 levels could be additionally reduced by translation inhibition through the PERK-eIF2 pathway in response to impaired maturation of Rh1 (see below). Conversely, an increase of *Arr1* and *Arr2* transcription was observed in the Retinitis Pigmentosa model *ninaE^G69D^/+* [70] that exhibits defective Rh1 maturation and low Rh1 levels but also excessive ER stress and age-dependent degeneration [71,72,73,74].

The second regulatory mechanism that underlies the about 2-fold decrease in molar abundances of the other phototransduction components (Figure 5B) appears to be post-transcriptional, since the transcription of the respective genes is not vitamin A-dependent [2]. While the decrease of inhibitory Arrestins upon impaired visual signaling due to low Rh1 levels appears intuitive, the reduction of phototransduction proteins that transduce and amplify the visual signal (e.g., Galphaq, NorpA; Figure 5B) or maintain the PI cycle is unexpected, since their decrease should additionally impair the speed and sensitivity of the visual response. Hence, it is possible that the previously reported visual defects of vitamin A-deficient flies that have been attributed to the dramatic loss of Rh1 were exacerbated by the reduced levels of major phototransduction components. Although the absolute molar decrease in phototransduction proteins is not proportional to the absolute decrease in Rh1 levels, this does not necessarily mean that the two defects are independent from one another. For instance, vitamin A deficiency causes the accumulation of immature Rh1 in the ER [13,14], which should result in ER stress [75] that could inhibit the translation of the highly expressed phototransduction proteins and lead to the preferential translation of stress-related proteins (see below). The same rationale could explain why the molar abundance of the highly expressed Chp protein is similarly reduced by vitamin A deficiency.

An alternative explanation for the comparable reduction of most phototransduction proteins in the vitamin A-deficient eye is that the substantially reduced rhabdomeres (Figure 3B) potentially cannot house the wild type amounts of phototransduction proteins, which might limit their production or cause their degradation. However, rhabdomere size defects do not necessarily cause lower phototransduction protein levels: for instance, *crb* mutants exhibit severe rhabdomere defects [76], but do not show changes in the molar abundances of phototransduction proteins [32]. 

### 4.2. Vitamin A Deficiency Affects Non-Vision-Specific Pathways of the Eye Proteome

Our head transcriptome [2] and eye proteome analysis (this study) revealed that there are *Drosophila* genes and proteins that strongly respond to vitamin A deficiency but are not part of the visual signaling cascade. For instance, vitamin A deprivation altered components of the translation machinery, which would be consistent with translation inhibition as the result of an unfolded protein response [75] elicited by impaired Rh1 maturation in photoreceptor neurons. Indeed, aminoacyl-tRNA synthetases are induced downstream of the PERK branch of the unfolded protein response [77,78], and we identified four aminoacyl-tRNA synthetase genes (*GluProRS*, *IleRS*, *LeuRS*, and *LysRS*) [2] and seven aminoacyl-tRNA synthetases (LeuRS, LysRS, MetRS, ThrRS, HisRS, IleRS, ValRS) that showed increased expression levels in response to vitamin A deficiency (Figure 4A). Since the PERK pathway suppresses general mRNA translation by phosphorylating eIF2 [73], this mechanism would also explain why we observed a reduction of photoreceptor proteins such as Rh1 and phototransduction proteins in vitamin A-deficient eyes. Moreover, *IleRS* and *LeuRS* genes, which were identified in both the vitamin A-dependent transcriptome and proteome, are also upregulated in the *ninaE^G69D^*/+ Retinitis Pigmentosa model that shows impaired Rh1 maturation, excessive ER stress, and low Rh1 levels [71,72,73]. Furthermore, we detected an increase in seven heat shock proteins and chaperones that included Cnx99A, an ER chaperone that is essential for Rh1 maturation [79]. Likewise, in mammals, vitamin A deficiency has been reported to cause unfolded protein responses and proteasome overloading due to the accumulation of immature opsin [80,81]. Further insights into the potentially conserved mechanisms could be gained from the comparison of the phototransduction protein, chaperone, and aminoacyl-tRNA synthetase abundances in Rh1 folding-deficient mutants that exhibit low Rh1 levels [82].

Our proteome analysis enriched vitamin A-dependent pathways whose mechanistic link to vitamin A deficiency remains to be elucidated. For instance, vitamin A deprivation decreased the subunits of major mitochondrial proteins (Figure 6) such as the SdhA subunit of the succinate dehydrogenase (Sdh) that acts in both the TCA cycle and the electron transport chain (as part of Complex II, Figure 6). Since the mitochondrial respiratory complexes consist of several subunits, an improper stoichiometry could reduce the mitochondrial productivity or generate harmful products such as reactive oxygen species. *SdhA* mutations have been linked to the progressive neurodegenerative disorder Leigh syndrome and the promotion of Sdh activity can prevent neurodegeneration in *Drosophila* [83,84,85]. Notably, TCA cycle intermediates are also reduced in a preclinical Retinitis Pigmentosa model and increased citrate production has a therapeutic effect against photoreceptor death [86]. Remarkably, vitamin A (retinol) serves in mammalian mitochondria as an essential cofactor of PKCdelta [87,88] in a complex with cytochrome c that stimulates pyruvate dehydrogenase [89], which converts pyruvate to Acetyl-CoA (Figure 6). This vitamin A-dependent mechanism regulates the final step of glycolysis and thus appears to have a different metabolic target than the mitochondrial proteins that we identified in the *Drosophila* eye (this study). Nevertheless, the vitamin A binding sites in PKC’s activation domain are conserved in *Drosophila* [90], and it is possible that both fly and mammalian mitochondria use the essential nutrient vitamin A as an obligate component of an energy homeostasis regulator complex [68,87], whose depletion causes oxidative stress and energy deprivation [91].

Lastly, we identified an increase in cuticle- and chitin binding-related proteins in the vitamin A-deficient eye. An intriguing question is whether the abundance increase of *Drosophila* lens and cuticular proteins is related to the drying and clouding of the cornea in vitamin A-deficient human eyes [1,92], and whether the latter shows a comparable increase in expression of cornea or lens proteins.

In conclusion, the molecular response to vitamin A deficiency alters components of both vision-specific and non-vision-specific pathways of the ocular proteome (Figure 7), which strongly suggests that vitamin A has functions beyond visual signaling in the *Drosophila* eye. Our study identified candidate molecules and pathways whose function in response to environmental or genetic stresses can now be studied using the powerful *Drosophila* toolkit.

## Figures and Tables

**Table 1:** Pathways and their protein components that respond to vitamin A deficiency in the *Drosophila* eye. For details, see text.

Pathways	Components
Phototransduction	NinaE/Rh1, Galphaq, Arr1, Arr2, InaC, InaD, NorpA, Trp
Phosphatidylinositol signaling	NorpA, PI4KIIIalpha, Ttc7, InaC, Ipp, Mtmr6
Citrate/TCA cycle	ScsbetaA, SdhA, Kdn, ATPsynbeta, Men-b
Genetic information processing	Hsp22, Hsp23, Hsc70-3, Cnx99A, Gp93, CCT5, Wbl
Transport and catabolism	Npc1a, Gga, Ppt1, Rb
Metabolism of proteins	LeuRS, LysRS, MetRS, ThrRS, HisRS, IleRS, ValRS

## Data Availability

Raw data are available upon request.

## Supplementary Materials

The following supporting information can be downloaded at: https://www.mdpi.com/article/10.3390/biom12081083/s1, Table S1: Analysis of lipidome, proteome, and pathways of vitamin A replete (M1) vs. vitamin A deficient (M0) retinas; Table S2: Label-free and MS Western quantification of phototransduction proteins; Table S3: Experimental settings for proteomics measurements.
