# Supplementary material for: Vitamin A Deficiency Alters the Phototransduction Machinery and Distinct Non-Vision-Specific Pathways in the Drosophila Eye Proteome
Source: Biomolecules. 2022 Aug 6;12(8):1083. doi: 10.3390/biom12081083 (PMC9405971; doi:10.3390/biom12081083)
Supplement: Supplementary file 1 [file biomolecules-12-01083-s001.zip › Supplementary Table S2 and S3.pdf]

Supplementary Table S2. Label-free and MS Western quantification of phototransduction proteins. For details, see text.

Label-free quantification			MS Western quantification	
**Protein**	**Fold change**	**p-value**	**Fold change**	**p-value**
NinaE/Rh1	12.96074	0.000200554	12.40209	0.003326
Arr2	1.624398	4.10241E-05	2.023933	0.004485
Arr1	1.237314	1016.791826	1.255242	0.02042
Galphaq	1.402132	0.201335377	1.718395	0.004184
NorpA	1.455259	0.002090038	2.318734	0.003052
Trp	1.257447	0.325019174	2.814573	0.133038
NinaC	1.227212	39.02052206	1.866455	0.006664
InaC	1.446729	1.27234E-05		
InaD	1.483609	3.46331E-05		
Rtp	1.651171	0.000879993		

Supplementary Table S3. Experimental settings for proteomics measurements.

**Parameter**	**Value**
**Full scan (MS1)**	
Scan range, m/z	350-1700
Microscans	1
Resolution @ m/z 200; FWHM	60,000
Lock mass, m/z (Siloxane)	445.120025
Data acquisition mode	Profile
Threshold intensity	3×10^{6}
Maximum IT, ms	50
**dd-MS2**	
Microscans	1

Resolution@ m/z 200; FWHM	15,000
Data acquisition mode	Centroid
AGC target	3×10^{6}
Maximum IT, ms	30
TopN (no msx)	12
Isolation window, m/z	1.6
Isolation offset, m/z	0.0
Fixed starting mass, m/z	140
NCE (no stepped NCE)	25%
Apex trigger Off	Off
Charge exclusion	Unassigned, 1 and above 8
Peptide match	Off
Exclude isotopes	On
Dynamic exclusion	30s
